
JOURNAL INFORMATION
==============================
NLM Title Abbreviation: ScientificWorldJournal
Journal ID: ScientificWorldJournal
NLM Journal ID: 101131163
Journal ID: TSWJ

The Scientific World Journal

ISSN: 2356-6140
EISSN: 1537-744X
Publisher: Wiley

ARTICLE INFORMATION
==============================
PMCID: PMC5936490
PMID: 15702222
DOI: 10.1100/tsw.2005.17
Article ID: 590165
Article version: 1
Subjects: Short Communication

Solitary Endobronchial Lymphangioma: A Case Report and Review of Literature

Chikkamuniyappa Shylashree *
Heim-Hall Josefine
Jagirdar Jaishree
UTHSCSA-Pathology, 7703 Floyd Curl Drive, San Antonio, TX 78229, USA
*Shylashree Chikkamuniyappa: chikkamuniya@uthscsa.edu
Publication date: 2005
Electronic publication date: 2005 Jan 28
Volume: 5
First page: 103
Last page: 108
Received 2004 Sep 20; Revised 2005 Jan 13; Accepted 2005 Jan 17
Copyright: Copyright © 2005 Shylashree Chikkamuniyappa et al.
Copyright year: 2005
License: This is an open access article distributed under the Creative Commons Attribution License, which permits unrestricted use, distribution, and reproduction in any medium, provided the original work is properly cited.
License URL: https://creativecommons.org/licenses/by/3.0/

Keywords: endobronchial, lymphangioma, adenoma, lung

==============================
Short Communication
*TheScientificWorldJOURNAL* (2005) 5, 103–108
ISSN 1537-744X; DOI 10.1100/tsw.2005.17

### Solitary Endobronchial Lymphangioma: A Case Report and Review of Literature

Shylashree Chikkamuniyappa*, Josefine Heim-Hall, and Jaishree Jagirdar
*UTHSCSA – Pathology, 7703 Floyd Curl Drive, San Antonio, TX 78229*

**E-mail:** chikkamuniya@uthscsa.edu

*Received September 20, 2004; Revised January 13, 2005; Accepted January 17, 2005; Published January 28, 2005*

**KEYWORDS:** endobronchial, lymphangioma, adenoma, lung

**DOMAIN:** pathology

### INTRODUCTION

Lymphangioma of the lung is extremely rare and few cases have been reported in the literature. Those described are located peripherally, are usually subpleural, and are cystic. The term “lymphangioma” refers to a benign collection of lymphatics that become sequestered from the normal lymphatic system during early embryogenesis[1]. They progressively grow from lymph accumulation and undergo cystic change in response to localized infection. Besides being interesting in terms of the location and appearance, it is an important differential diagnosis to be kept in mind by pathologists, radiologists, and bronchoscopists alike.

### REPORT OF A CASE

A 56-year-old Hispanic female presented with shortness of breath. A 2.5-cm, well-circumscribed mass was identified on a chest radiograph. The lesion had been followed with serial CT examinations over the past 6 months and a slight increase in size had been noted. Mediastinal lymphadenopathy was also seen on CT scans. The patient had a known history of systemic hypertension and Type II insulin-dependent diabetes. She is a nonsmoker and nondrinker.

Radiologically, the nodule was perfectly spherical, had a sharp border (Fig. 1), and pushed the lung tissue aside suggesting a slow, uniform growth rate rather than an aggressive process[2]. On CT scan, the nodule demonstrated a homogenous signal that was of lower intensity than the chest wall musculature, implying fat or mucus. Differential diagnosis at this point included granuloma, hamartoma, arteriovenous malformation, and intrapulmonary bronchogenic cyst. However, a malignant process could not be entirely excluded, so the lesion was excised. Given the proximity of the nodule to a major pulmonary vein, a lobectomy rather than a wedge resection was performed. Hilar and subcarinal lymph nodes were sampled. On a 6-month follow-up, the patient was doing fine.

*Corresponding author.
©2005 with author.

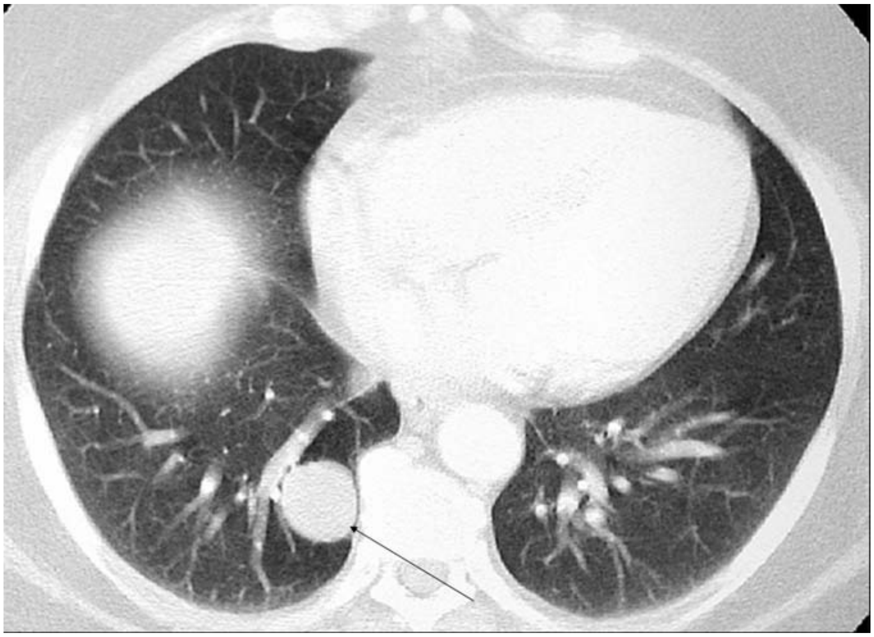

**FIGURE 1.**

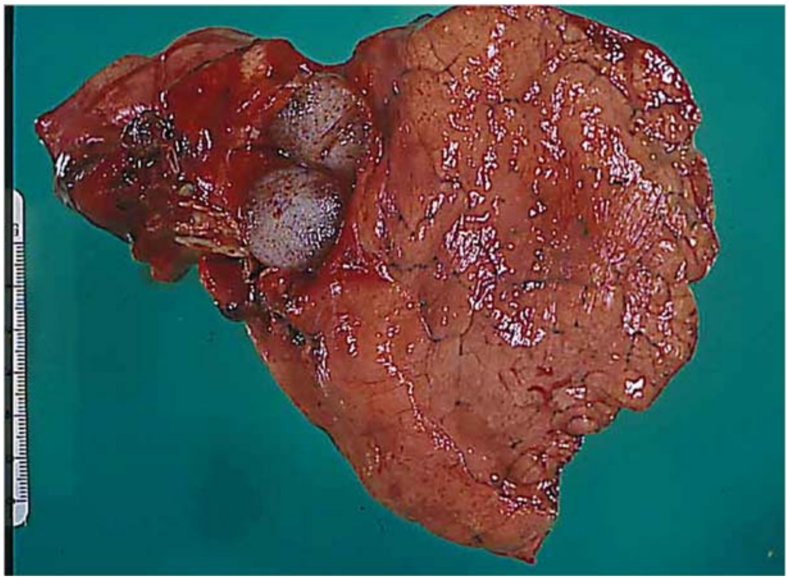

**FIGURE 2.**

### PATHOLOGIC FINDINGS

### Gross Pathology

A well-defined tumor nodule was identified arising from the main bronchus measuring 2.5 cm in greatest diameter. It was located in the right lower lobe, 3.5 cm from the bronchial margin. The tumor was sessile, white, soft, glistening, and mucoid on cut surface (Fig. 2). It displayed finely reticulated vessels over its surface. The adjacent parenchyma was somewhat compressed, but otherwise well aerated.

### Microscopic Pathology

The lesion consisted of cystic spaces of varying sizes separated by myxoid/collagenous, paucicellular septa. They were arranged in a honeycomb pattern similar to pulmonary alveoli (Fig. 3). The cystic spaces were lined by flat, inconspicuous cells that stained with CD31 (Fig. 4) and focally with CD34. In some areas, eosinophilic, cuboidal, epithelial cells were present. The lumina contained wispy, eosinophilic material. No red cells were seen in the spaces. The adjacent lung parenchyma was flat and compressed. Immunohistochemical stains cytokeratin (AE1/3) and TTF-1 were negative. All mediastinal lymph nodes sampled had well-formed, noncaseating, sarcoid-type granulomas. Special stains for organisms were negative.

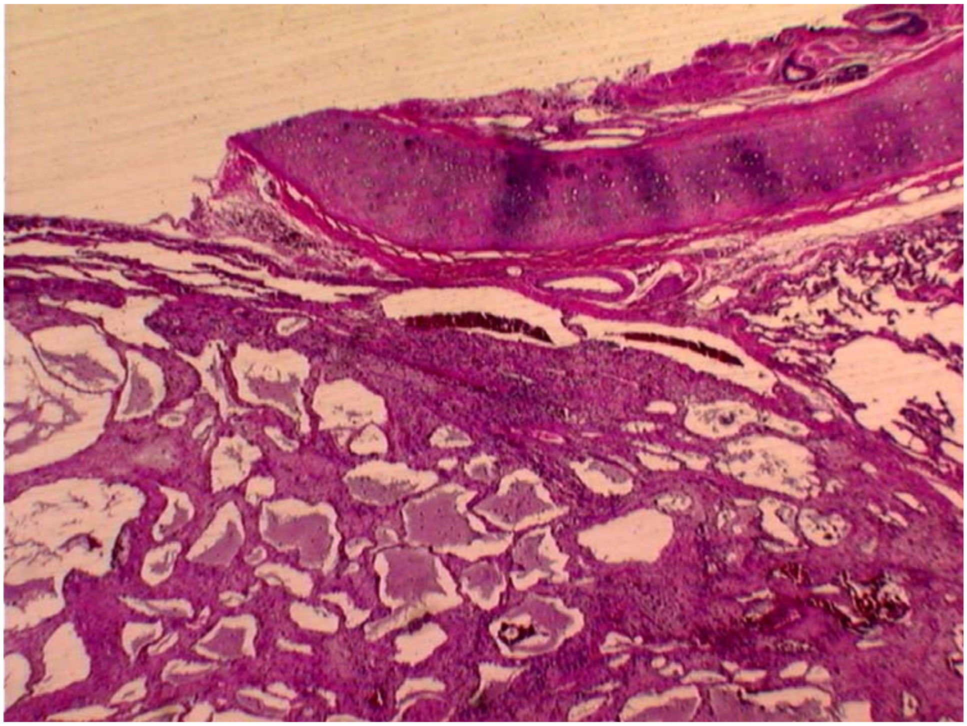

**FIGURE 3.**

### COMMENT

We present an unusual case of a solitary lymphangioma of the lung that was endobronchial, gross, and had microscopic appearance that mimicked a mucus gland adenoma of the bronchus. It did not contain lymphocytes. It was only after immunohistochemical stains that the true nature of the lesion was apparent. The first case of a pulmonary lymphangioma was reported by Wada et al.[3] in 1974. This lesion was subpleural and the periphery of the lesion was separated by a thin, fibrous, connective tissue and sections also showed associated lymphoid tissue in the interstitium. At that time, there was some controversy whether or not lymphangioma was a true tumor or hamartoma or malformation. Hillard et al.[4] emphasized that lymphangioma is a part of a spectrum of developmental lymphatic disorders that share a common pathogenesis and Brown et al.[5] confirmed the common origin by immunohistochemical studies.

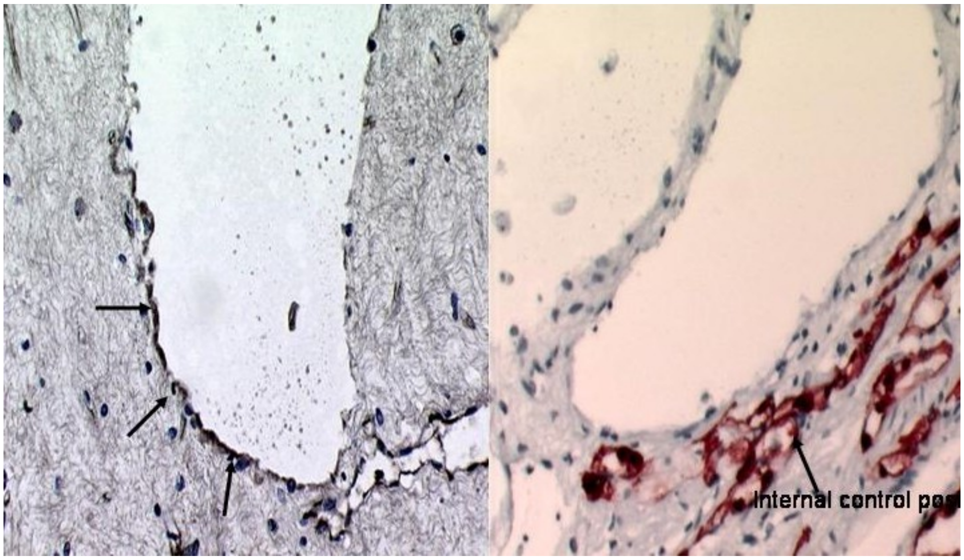

**FIGURE 4.**

The exact incidence of these rare tumors is not known. They occur both in children and in adults and have been noted to be more frequent in women. They are frequently associated with hemangiomas[6]. They usually present as solitary cystic lesions peripherally. They vary in size. Grossly, they are cystic or spongy. Symptoms vary widely depending on the location, size, patient's age, and extent of disease. In neonates and infants, cystic lymphangiomas often present with pneumothorax and respiratory distress. In adults, they are often asymptomatic, but may not always follow an innocuous course. The CT appearance and MRI features vary, but help characterize the possible contents of the nodule and define its anatomic relationships. The most common appearance is that of a cystic mass with smooth margins displaying calcifications and spiculations.

Surgical resection should be considered in light of the neoplastic nature. Optimal treatment is resection of the tumor with sparing of the lung parenchyma. A wedge resection may be sufficient in some cases. Minimally invasive, thoracoscopic techniques have also been tried. Occasionally, lobectomy is required when a more limited surgery is not feasible or when it is difficult to clinically differentiate this lesion from a primary lung cancer. Recurrences are extremely rare.

Important mimickers in this location to be considered are mucous gland adenoma, bronchial carcinoid tumor, alveolar adenoma, mucinous tumors, mucoepidermoid carcinoma, inflammatory masses, hamartoma, and endobronchial hemangioma.

- Mucus gland adenoma of the bronchus displays several histologic patterns, but the most characteristic feature is the presence of cystic, mucus-filled glands protruding into the bronchial lumen. These glands may be lined by cytologically benign, columnar, oncocytic, cuboidal, or flattened mucinous cells. Small tubular glands or papillary structures may be present. The fluid filling these cysts is usually a dense, colloid-like material. Histochemically, mucicarmine, neutral and acid mucins, alcian blue, and PAS stains are positive. The tumors are positive for epithelial markers by immunohistochemistry[7].
- Bronchial carcinoids are well-circumscribed, largely endobronchial, polypoid masses with typically a yellowish cut surface. They are characterized by an organoid growth pattern and uniform cytologic features consisting of moderate cytoplasm and finely granular chromatin[8].
- Alveolar adenoma is a multicystic, benign neoplasm that recapitulates alveolar epithelium and mesenchyme. The cystic spaces are lined by type II pneumocytes. The tumor is solitary, circumscribed, gray/white, and not gelatinous like the current lesion and situated in the periphery of the lung. The neoplastic pneumocytes are typically TTF-1 positive. The present lesion was TTF-1 negative ruling out an alveolar adenoma.

- Mucinous cystadenoma is a unilocular, cystic lesion lined by benign, columnar, mucinous epithelium. Borderline mucinous tumors are also primarily unilocular, noninvasive, and lined by atypical, columnar, mucinous epithelium displaying stratification, nuclear pleomorphism, and hyperchromatism. Mucinous cystadenocarcinoma is invasive and has solid areas in addition to the mucinous, columnar epithelium.
- Mucoepidermoid carcinomas are rare tumors presenting as a polypoid, pink, endobronchial nodule with a glistening, mucoid cut surface. Microscopically, it has a variable population of mucin-secreting, squamous, and intermediate cells.
- Hamartomas are the most common benign tumors of the lung. Grossly, they have a firm, marble-like consistency. They consist of haphazardly organized mature cells and tissues. They are composed mostly of masses of hyaline cartilage with a myxoid connective tissue, adipose cells, smooth cells, and clefts lined by respiratory epithelium.
- Endobronchial hemangiomas are well-circumscribed, reddish, hypervascular lesions that are rare.[9] The tumors are characterized by large cavernous or small capillary vessels and reveal a strong staining for vimentin and CD34.

In summary, a rare case of a solitary pulmonary lymphangioma is presented that closely mimicked a mucus gland adenoma of the bronchus. Immunohistochemistry was useful in arriving at the correct diagnosis.

### REFERENCES

1. Drut, R. and Mosca, H.H. (1996) Intrapulmonary cystic lymphangioma. *Pediatr. Pulmonol.* **22**, 204–206.
2. Zwirewich, C.V., Vedal, S., Miller, R.R., et al. (1991) Solitary pulmonary nodule: high-resolution CT and radiologic-pathologic correlation. *Radiology* **179**, 469–476.
3. Wada, A., Taeishi, R., Terazawa, T., Matsuda, M., and Hattori, S. (1974) Lymphangioma of the lung. *Arch. Pathol.* **98**, 211–213.
4. Hillard, R.I., Mckendry, J.B.J., and Phillips, M.J. (1990) Congenital abnormalities of the lymphatic system: a new clinical classification. *Pediatrics* **86**, 988–994.
5. Brown, M., Pysher, T., and Coffin, C.M. (1999) Lymphangioma and congenital pulmonary lymphangiectasis: a histologic, immunohistochemical and clinicopathologic comparison. *Mod. Pathol.* **12**, 569–575.
6. Al-Hilli, F. (1987) Lymphangioma (or alveolar adenoma?) of the lung. *Histopathology* **11,** 979–980.
7. England, D.M. and Hochholzer, L. (1995) Truly benign “bronchial adenoma”: report of 10 cases of mucous gland adenoma with immunohistochemical and ultrastructural findings. *Am. J. Surg. Pathol.* **19**, 887–899.
8. Du, E.Z., Goldstraw, P., Zacharias, J., et al. (2004) TTF-1 expression is specific for lung primary in typical and atypical carcinoids: TTF-1-positive carcinoids are predominantly in peripheral location. *Hum. Pathol.* **35(7),** 825–831.
9. Bowyer, J.J. and Sheppard, M. (1990) Capillary hemangioma presenting as a lung pseudocyst. *Arch. Dis. Child.* **65(10),** 1162–1164.

---

**This article should be referenced as follows:**

Chikkamuniyappa, S., Heim-Hall, J., and Jagirdar, J. (2005) Solitary endobronchial lymphangioma: a case report and review of literature. *TheScientificWorldJOURNAL* **5**, 103–108.

**Handling Editor:**

K. Kayser, Principal Editor for *Pathology* and Associate Editor for *Imaging* — domains of *TheScientificWorldJOURNAL*.

---

### BIOSKETCH

**Shylashree Chikkamuniyappa** is a fifth year anatomic/clinical resident and hematopathology fellow at the University of Texas Health Science Center in San Antonio. He received his medical training in Bangalore, India. Dr. Chikkamuniyappa was actively involved in the racemase and 5-alpha reductase studies on prostate cancer at his institution and is currently involved in projects studying diffuse large B-cell lymphoma and post-transplant lymphoproliferative disorders.
